# Supplementary material for: Investigation of Extracted Plasma Cell-Free DNA as a Biomarker in Foals with Sepsis
Source: Vet Sci. 2024 Aug 1;11(8):346. doi: 10.3390/vetsci11080346 (PMC11359113; doi:10.3390/vetsci11080346)
Supplement: Supplementary file 1 [file vetsci-11-00346-s001.zip › vetsci-3099843-supplementary.pdf]

Table S1: Demographics Table. FPT=Failure of passive transfer of immunity, NMS=neonatal maladjustment syndrome.

Case Number	Breed	Gender	Age Hours	Extracted cfDNA Concentration	Neat CfDNA Concentration	Assigned Group	Diagnosis	Blood Culture	Sepsis Score	Neutrophil Count	Survival
20-01	Quarter Horse	Filly	32h	35.94	472.7	Healthy	Healthy	Negative	5	2.3	Yes
20-02	Thoroughbred	Colt	36h	13.54	432	Healthy	Healthy	Negative	0	6.24	Yes
20-03	Quarter Horse	Colt	32h	22.6	404.7	Healthy	Healthy	Negative	0	4.29	Yes
20-05	Thoroughbred	Colt	60h	115.2	613	Healthy	Healthy	Negative	1	11.76	Yes
20-07	Thoroughbred	Filly	40h	30.4	395.33	Healthy	Healthy	Negative	4	6.34	Yes
20-13	Thoroughbred	Colt	50h	31.34	720.6	Healthy	Healthy	Negative	2	6.32	Yes
20-15	Thoroughbred	Colt	33h	19.98	295	Healthy	Healthy	Negative	3	6.02	Yes
20-16	Thoroughbred	Filly	24h	5.28	432.6	Healthy	Healthy	Negative	0	4.15	Yes
20-18	Percheron	Colt	28h	55.32	411	Healthy	Healthy	Negative	2	6.34	Yes
20-19	Quarter Ho	Filly	60h	114.54	635.3	Healthy	Healthy	Negative	0	6.16	Yes
20-14	Thoroughbred	Colt	36h	26	541	Healthy	Healthy	Staph chromogenes (contamination)	0	6.16	Yes
20-30	Warmblood	Colt	80h	358.14	822	Healthy	Healthy	Negative	0	5.7	Yes

22-03	Quarter Horse	Filly	28h	83.2	71	Healthy	Healthy	Negative	0	6.02	Yes
22-04	Quarter Horse	Filly	28h	90	51.8	Healthy	Healthy	Negative	0	3.1	Yes
22-05	Quarter Horse	Filly	28h	99.2	55.4	Healthy	Healthy	Negative	0	6.1	Yes
20-24	Thoroughbred	Filly	35h	37.06	518.7	Healthy	Healthy	Negative	5	4.73	Yes
20-27	Thoroughbred	Colt	28h	277.6	573.3	Healthy	Healthy	Negative	3	4.9	Yes
21-05	Thoroughbred	Colt	24h	83.6	336	Healthy	Healthy	Negative	4	6.61	Yes
20-21	Paint	Colt	36h	10.5	434	Healthy	Healthy	Negative	5	Unknown	Yes
20-25	Quarter Horse	Colt	31h	199.6	537.3	Healthy	Healthy	Negative	2	Unknown	Yes
20-08	Thoroughbred	Colt	24h	93.06	589.3	Healthy	Healthy	Negative	0	6.93	Yes
21-02-I	Quarter Horse	Unknown	24h	125	30.4	Healthy	Healthy	Not Submitted	0	4.17	Yes
20-12	Thoroughbred	Colt	31h	26.6	466	Healthy	Healthy	Staph equorum (contamination)	0	11.76	Yes
22-17	Unknown	Filly	24h	94	20.4	Sick Not Septic	FPT	Negative	3	3.34	Yes
22-12	Appaloosa	Colt	20h	78.4	226	Sick Not Septic	FPT	Negative	9	14.84	Yes
22-14	Unknown	Unknown	24h	86.4	29.4	Sick Not Septic	NMS	Negative	8	7.6	Yes
22-06	Quarter Horse	Colt	24h	78.4	52.4	Sick Not Septic	NMS	Negative	10	9.5	Yes
21-10	Fjord	Filly	24h	88.6	62.4	Sick Not Septic	Diarrhea	Negative	7	6.059	Yes

23-13	Quarter Horse	Colt	12h	57.2	565	Sick Not Septic	FPT	Staph (Contamination)	7	6.95	Yes
23-10	Warmblood	Filly	24h	3.16	298	Sick Not Septic	Diarrhea	Negative	7	2.47	Yes
23-H3	Thoroughbred	Filly	24h	3.06	968	Sick Not Septic	Meconium Impaction	Negative	7	9.4	Yes
23-H5	Thoroughbred	Colt	24h	2.92	677	Sick Not Septic	FPT	Negative	8	5	Yes
S11	Thoroughbred	Colt	48h	18.02	499	Sick Not Septic	Diarrhea	Negative	6	2.51	Yes
S12	Percheron	Filly	1hr	29.5	573	Sick Not Septic	Meconium Impaction	Negative	7	5.04	Yes
S19	Quarter Horse	Colt	0h	129.8	737	Sick Not Septic	NMS	Negative	7	5.01	Yes
S20	Quarter Horse	Filly	0h	21	487	Sick Not Septic	NMS	Negative	6	4.25	Yes
S21	Thoroughbred	Filly	0h	12.74	596	Sick Not Septic	FPT	Negative	13	7.24	Yes
S24	Belgian	Filly	48hr	13.3	372	Sick Not Septic	Colic	Negative	7	4.05	Yes
S6	Quarter Horse	Colt	240h	297.4	878	Sick Not Septic	Pneumonia	Negative	7	8.47	Yes
22-01-ISU	Gypsy Vanner	Colt	24h	106.4	56.4	Sick Not Septic	NMS	Negative	0	4.64	Yes
20-23	Paint	Colt	24h	430.66	738.7	Sick Not Septic	FPT	Negative	5	3.34	Yes
22-15	Unknown	Unknown	24h	103.2	115	Sick Not Septic	NMS	Negative	2	4	Yes
20-34	Quarter Horse	Filly	24h	33.2	322	Sick Not Septic	FPT	Not Submitted	7	6.63	Yes

20-04	Thoroughbred	Colt	60h	562.66	997.3	Septic	Pneumonia	Staph chromogenes, on Gentamicin	6	6.63	Yes
20-06	Thoroughbred	Filly	24h	69.6	373	Septic	Diarrhea	Heavy, bacillus sp.	0	8.7	Yes
20-33	POA	Filly	40h	175.06	572	Septic	Diarrhea	E. coli	12	4.15	No
20-10	Thoroughbred	Colt	68h	76.4	405	Septic	Sepsis	Strep alph hemoly. received penicillin	1	6.16	Yes
20-11	Standardbred	Filly	60h	22.2	418	Septic	Diarrhea	Staph chromogenes	7	12.04	Yes
20-29	Standardbred	Filly	24h	141.34	607.3	Septic	Diarrhea	Negative	20	7.221	No
20-32	POA	Filly	72h	15.6	526.6	Septic	Prematurity	Not Submitted	13	6.34	Yes
20-20	Thoroughbred	Colt	120h	26.06	447.3	Septic	Sepsis	E. coli	11	5.89	Yes
20-26	Thoroughbred	Colt	72h	89.46	416	Septic	FPT	Pseudomonas, gram positive rod	11	3.56	Yes
22-07	Quarter Horse	Filly	36h	113.2	37.2	Septic	Sepsis	Gram positive (not listed)	5	Unknown	Yes
20-28	Paint	Filly	72h	34.26	482	Septic	Diarrhea	Actinobacillus sp.	4	3.05	Yes
22-11	Irish Hunter	Filly	72h	87.6	252	Septic	Prematurity	Aero	4	Unknown	Yes
23-06	Percheron	Filly	72h	12.04	409	Septic	Pneumonia	Negative	19	Unknown	Yes
23-14	Quarter Horse	Filly	120h	63.2	568	Septic	Sepsis	E.Coli	13	13.2	No
23-15	Mini	Colt	96h	8.64	458	Septic	Sepsis	Staph	5	4.189	Yes
23-09	Saddlebred	Filly	24h	10.06	444	Septic	FPT	Coryn/Steo	7	0.146	Yes
23-18	Quarter Horse	Colt	48h	6.44	470	Septic	Pneumonia	Cocci	12	0.92	No

Table S2: Extracted cfDNA concentrations before and after treatment with Turbo DNase. (% degraded = ((Extracted cfDNA - turbo DNA treated)/Extracted cfDNA) x 100.)

Sample	Extracted cfDNA before Turbo DNase (ng/mL)	Extracted cfDNA after Turbo DNase (ng/mL)	% degraded
1	56	0	100%
2	106	0	100%
3	28.6	0	100%
4	168	0	100%
5	23.8	0	100%

Table S3. Neutrophil counts in 55 hospitalized neonatal foals. SS=sepsis score. (Blood culture criteria excluded.)

Foal Category	Median (range) neutrophil count ($x10^3$cells/uL)	95% confidence interval ($x10^3$cells/uL)
Healthy (SS≤5) (n=21)	6.02 (2.3 to 11.76)	4.22 to 6.34
Sick non-septic (SS 6-11) (n=17)	5.04 (2.47 to 14.84)	4.15 to 8.035
Septic (SS ≥12) (n=17)	6.16 (0.146 to 13.2)	3.855 to 7.961
